# Integrating Multivariate Analysis and DNA Barcoding for Amaranth Germplasm Characterization and Promising Genotype Selection

**DOI:** 10.3390/plants15101493

**Published:** 2026-05-13

**Authors:** Adnan Kanbar, Yaman Jabbour, Peter Nick

**Affiliations:** 1 National Unit for Environmental Research and Services (NUERS), Research Sector, Kuwait University, P.O. Box 5969, Al-Shadadiya 13060, Kuwait; 2 Department of Field Crops, Faculty of Agriculture, University of Damascus, Damascus P.O. Box 30621, Syria; 3 Field Crop Department, Aleppo Research Center, General Commission for Scientific Agriculture Research (GCSAR), Damascus P.O. Box 12573, Syria; yaman.jab@gmail.com; 4 Molecular Cell Biology, Joseph Kölreuter Institute for Plant Sciences, Karlsruhe Institute of Technology, Fritz-Haber-Weg 4, 76131 Karlsruhe, Germany

**Keywords:** *Amaranthus*, genetic diversity, heritability, path coefficient, Mahalanobis D^2^, multivariate statistics, *psbA-trnH*, ITS marker

## Abstract

Amaranth (*Amaranthus* spp.) is a multifaceted genus of C4 plants with significant nutritional and agronomic potential, yet it remains underutilized in mainstream agriculture. Despite growing interest in Amaranth, most germplasm studies have used either phenotypic or molecular approaches alone, lacking integration. Multivariate methods have not been systematically applied to identify promising genotypes, and species-specific selection indices for grain Amaranth remain unexplored. To address these gaps, this study comprehensively characterized 84 Amaranth genotypes representing multiple species (*A. caudatus*, *A. cruentus*, *A. hypochondriacus*, *A. hybridus*, *A. spinosus*, *A. powellii*, *A. tricolor*, and 38 accessions of unknown taxonomic status) using field experiments in a randomized complete block design with three replications and DNA barcoding with chloroplast (*psbA-trnH*) and nuclear (ITS) markers. Analysis of variance revealed highly significant differences (*p* < 0.01) among genotypes for all six agronomic traits evaluated, confirming substantial genetic variability with grain yield exhibiting the widest variation (CV = 28.55%), ranging from 0.25 to 125.56 g/plant. High broad-sense heritability estimates (0.79–0.99) coupled with high genetic advance, particularly for grain yield (117.54%), indicated that these traits would respond favorably to selection. Path analysis and stepwise regression identified early flowering, long inflorescences, and heavy seeds as the primary determinants of grain yield, collectively explaining 27% of yield variation. Mahalanobis D^2^ analysis identified nine multivariate outliers with distinct phenotypic profiles, among which G39 emerged as the most promising breeding candidate, combining exceptional yield (90.50 g/plant) with desirable architecture, long inflorescence, and large seeds. Principal component analysis further resolved trait complexes, identifying 11 PC1-selected promising genotypes as donors for plant architecture and three PC2-selected promising genotypes as donors for seed size characteristics. Molecular analysis revealed distinct genetic relationships. *A. caudatus* (*kiwicha*) exhibited limited haplotype diversity indicating a narrow genetic base, while *A. cruentus* and *A. hypochondriacus* showed broader diversity, with the nuclear ITS network providing clearer resolution than chloroplast markers due to biparental inheritance. Outlier genotypes, including G82, G83, G13, G10, and G39, occupied unique haplotype positions, confirming that their phenotypic distinctiveness corresponds to genuine genetic differentiation. The novelty of this study lies in integrating multivariate biostatistical techniques (heritability, path analysis, Mahalanobis D^2^, PCA, and stepwise regression) with two complementary DNA barcode systems (chloroplast and nuclear) within a single germplasm collection. This integrated approach provides breeders with well-characterized germplasm, validated selection criteria, and prioritized parental materials for Amaranth improvement. Further multi-location and multi-season evaluations are recommended to ensure the stability and adaptability of these promising germplasm accessions.

## 1. Introduction

Amaranth (*Amaranthus* spp.) is a multifaceted genus of C4 plants that has served humanity for millennia as both a leafy vegetable and a pseudo-cereal grain. Originating in the Americas, where it was a staple food of pre-Columbian civilizations including the Aztecs and Incas, Amaranth has since spread to diverse agroecological regions across Africa, Asia, and Europe [1,2]. The genus encompasses approximately 60–70 species, including three principal grain Amaranths (*A. caudatus*, *A. cruentus*, and *A. hypochondriacus*) and numerous leafy vegetable types such as *A. tricolor*, *A. viridis*, and *A. dubius* [3,4]. These three grain species represent the primary Amaranth types utilized for food production globally and are now cultivated across diverse geographical regions spanning North America (USA and Canada), South America (Guatemala, Peru, and Argentina), Europe (Germany and France), Asia (India and China), and Africa (Ethiopia).

This remarkable geographic expansion reflects the crop’s exceptional adaptability to adverse environmental conditions, including tolerance to saline and alkaline soils, resilience to high temperatures, capacity to thrive at high altitudes, and ability to withstand prolonged periods of water scarcity [5,6,7]. Moreover, Amaranth qualifies as an environmentally sustainable crop due to its inherent pest resistance [8]. Despite its historical significance, nutritional excellence, and remarkable adaptability, Amaranth remains underutilized in mainstream agriculture, often relegated to the category of orphan crops grown in marginal areas by resource-limited farmers [9,10,11].

Amaranth exhibits remarkable morphological diversity across the genus, with plant forms ranging from 0.3 to 3.0 m in height and displaying extensive variation in growth habits (erect, prostrate, or decumbent), stem color polymorphism (green, red, purple, or striped), branching intensity, pubescence, leaf arrangement (alternate or opposite), and inflorescence architecture (terminal and axillary spikes, panicles, or glomerules differing in density, length, orientation, and color) [2,3,12]. Seed characteristics also contribute to this diversity, with colors spanning yellow, white, brown, black, and pink, alongside considerable interspecific variation in seed size and surface luster [1,12]. Phenological traits such as days to flowering and maturity show wide variation across the genus, ranging from early- to late-maturing types, enabling adaptation to diverse photoperiod regimes and growing seasons [13,14,15]. This extensive morphological and phenological plasticity, while valuable for adaptation to diverse environments across tropical, subtropical, and temperate regions, complicates taxonomic classification as characteristics such as pigmentation show wide segregation and plant size varies with environmental factors including sunlight hours, soil conditions, and cultural practices [14]. Furthermore, frequent hybridization between cultivated and weedy species, particularly with *A. hybridus*, produces intermediate morphological forms, challenging species identification based solely on morphological descriptors [6,8,16,17].

Amaranth is predominantly self-pollinated, yet it exhibits a notable rate of natural cross-pollination (approximately 20–30%) mediated by wind [8]. This relatively high outcrossing rate justifies the application of molecular markers alongside phenotypic evaluation to accurately assess genetic diversity and population structure within germplasm collections [18,19]. Furthermore, Amaranth is a warm-season C4 crop typically cultivated from spring to early summer. Its exceptional adaptability to increasing temperatures and water scarcity positions it as a promising crop for climate change mitigation and adaptation strategies [5,6]. The present study, therefore, provides a valuable assessment of Amaranth germplasm that could be utilized across diverse European agro-climatic zones, as well as in other regions facing increasing environmental stresses.

Despite these attributes, Amaranth breeding programs have lagged behind those of major cereals, with most cultivation still relying on landraces and limited systematic improvement [6]. Genetic resources conserved in gene banks remain largely uncharacterized and underutilized, yet their effective utilization requires comprehensive evaluation of phenotypic diversity, genetic parameters, and molecular relationships [19]. Traditional breeding relying on visual selection for one or two traits often fails to capture complex yield architecture. While indirect selection based on simple phenotypic correlations is common, this approach reveals only the magnitude of association without distinguishing between direct and indirect effects [20]. Path coefficient analysis addresses this limitation by partitioning correlation coefficients into direct and indirect effects, enabling identification of traits with genuine influence on yield [21]. Building on this foundation, multivariate approaches have become indispensable: Mahalanobis D^2^ analysis quantifies genetic divergence to identify distinct accessions for hybridization [22]; principal component analysis reduces dimensionality to reveal trait association patterns [23,24]; and stepwise regression selects influential traits for predicting yield, providing focused selection indices [25]. The integration of these techniques transforms complex trait interrelationships into actionable criteria for efficient genetic improvement.

While phenotypic characterization and multivariate analysis provide valuable insights into trait variation and relationships, they cannot fully capture the evolutionary history and genetic relatedness among accessions. This limitation has been addressed through the application of DNA barcoding, which utilizes standardized genetic markers to assess biodiversity and evolutionary relationships [19,26]. In plants, the combination of chloroplast and nuclear DNA sequences has proven particularly informative for resolving taxonomic uncertainties and tracing evolutionary trajectories [12,26,27,28]. Among the various analytical approaches for visualizing genetic relationships, median-joining networks constructed from DNA barcode sequences have emerged as powerful tools for depicting haplotype diversity and evolutionary connections [29]. Unlike traditional phylogenetic trees, which assume bifurcating branching patterns, network approaches accommodate the reality of population-level processes including incomplete lineage sorting, hybridization, and recombination, making them particularly suitable for closely related species and intraspecific studies [18,30]. These networks display unique haplotypes as circles proportional to their frequency, with mutational steps represented as connecting lines, effectively illustrating both genetic distances and evolutionary relationships among accessions.

The integration of molecular data with phenotypic and multivariate analyses creates a comprehensive framework: multivariate approaches identify phenotypically distinct genotypes, while molecular networks validate whether this distinctiveness corresponds to genetic differentiation. This synergy is particularly valuable in *Amaranthus*, where morphological plasticity and hybridization complicate taxonomy [6].

Accessions identified through both approaches that occupy unique haplotype positions while exhibiting superior agronomic performance represent prioritized materials for conservation and breeding. Additionally, clustering patterns of unknown accessions alongside reference species provide provisional taxonomic assignments. DNA barcoding thus serves not as a standalone tool but as a complement that reinforces insights gained from phenotypic characterization [26]. Several knowledge gaps remain in Amaranth germplasm characterization. First, most studies have used either phenotypic or molecular approaches alone, lacking an integrated framework. In those cases, where molecular phylogeny has been applied, this was based on statistically inferred distances only. However, the specific SNPs required to change a given genotype to its neighbor are informative by themselves (homologisation based on specific quality). This can be addressed by inferring haplotype networks. Second, multivariate methods have not been systematically applied to identify promising genotypes with both superior agronomic performance and unique genetic profiles. Third, species-specific selection indices for grain Amaranth are largely unexplored. The study addresses these gaps by integrating multivariate biostatistical techniques with chloroplast and nuclear DNA barcoding.

The central hypothesis of the present study is that genotypes exhibiting extreme phenotypic profiles (multivariate outliers) will occupy unique haplotype positions in molecular networks, indicating that phenotypic distinctiveness corresponds to genuine genetic differentiation rather than environmental plasticity alone. Unlike previous studies that used only phenotypic or molecular approaches [14,24], the current work integrates both approaches within a single germplasm collection to test this hypothesis.

Accordingly, we undertook to comprehensively characterize 84 Amaranth genotypes representing multiple species and diverse origins. The specific objectives were to: (i) assess the extent of phenotypic variation for six agronomic traits; (ii) estimate genetic parameters including heritability and genetic advance; (iii) analyze trait associations and identify direct and indirect effects on grain yield; (iv) identify multivariate outliers and promising genotypes using D^2^ and PCA approaches; and (v) evaluate genetic relationships using chloroplast and nuclear DNA barcodes. The integration of these complementary approaches aims to provide breeders with well-characterized germplasm, validated selection criteria, and prioritized parental materials for accelerated Amaranth improvement.

## 2. Results

### 2.1. Phenotypic Variation Among 84 Amaranth Genotypes

The analysis of variance for days to flowering, plant height, inflorescence length, grain yield, cross-sectional seed area, and 1000-seed weight (TSW) revealed highly significant differences (*p* < 0.01) among the 84 Amaranth genotypes (Table S1). Grain yield and 1000-seed weight data were square-root transformed prior to analysis to meet normality assumptions. These results indicate that sufficient genetic variability exists within the germplasm panel selected for this study. Block effects were significant only for days to flowering (*p* < 0.01), while remaining non-significant for all other traits, indicating that environmental heterogeneity within the experimental field had minimal influence on most agronomic traits.

Extensive phenotypic variation was observed among the 84 Amaranth genotypes for all six agronomic traits evaluated under irrigated conditions (Table 1; Figure S1). The descriptive statistics revealed wide ranges and substantial variability, indicating considerable genetic diversity within the germplasm panel. Days to flowering ranged from 68.0 to 101.0 days (mean = 80.8 days), with a low coefficient of variation (CV = 0.87%), indicating moderate consistency in flowering behavior across genotypes. Plant height varied from 80.0 cm to 295.0 cm (mean = 179.1 cm), with substantial diversity (CV = 4.91%), encompassing both dwarf and exceptionally tall accessions. Inflorescence length ranged from 10.0 cm to 170.0 cm (mean = 91.0 cm), displaying a CV of 8.74%. Grain yield exhibited the widest variation, ranging from 0.25 g plant^−1^ to 125.56 g plant^−1^ (mean = 30.1 g plant^−1^), with an exceptionally high CV of 28.55%, demonstrating extraordinary diversity in productivity potential.

Cross-sectional seed area ranged from 0.50 mm^2^ to 1.79 mm^2^ (mean = 1.22 mm^2^), with moderate variability (CV = 7.70%), while 1000-seed weight varied from 0.15 g to 1.11 g (mean = 0.69 g), with considerable variation (CV = 9.00%). The frequency distributions (Figure S1) showed continuous variation for all traits, with days to flowering and plant height exhibiting near-normal distributions (skewness = 0.72 and 0.44, respectively), while grain yield displayed positive skewness (0.83) and elevated kurtosis (4.37), indicating that while most genotypes produced moderate yields, a subset of high-yielding accessions contributed to the extended tail of the distribution. The wide ranges and CV values, particularly for grain yield (28.55%), inflorescence length (8.74%), and plant height (4.91%), confirm extensive genetic diversity within the germplasm, thereby providing significant scope for genetic improvement through selective breeding.

### 2.2. Genetic Parameters: Variance Components, Heritability, and Genetic Advance

Genetic parameter estimates revealed important insights into the inheritance patterns and selection potential of the six agronomic traits (Table 2). Phenotypic (σ^2^p) and genotypic (σ^2^g) variance were highest for plant height (σ^2^p = 2066.72, σ^2^g = 1989.25) and grain yield (σ^2^p = 431.18, σ^2^g = 357.12), indicating substantial genetic variability available for selection in these traits. The genotypic coefficient of variation (GCV) and phenotypic coefficient of variation (PCV) followed similar patterns across traits, with grain yield exhibiting the highest GCV (62.70%) and PCV (68.89%), thereby confirming exceptional genetic variability for productivity enhancement. Inflorescence length (GCV = 26.86%, PCV = 28.25%) and plant height (GCV = 24.90%, PCV = 25.38%) also showed high coefficients, while days to flowering displayed the lowest values (GCV = 9.83%, PCV = 9.87%). Minimal differences between GCV and PCV across all traits indicated that environmental influence on trait expression was limited, consistent with the ANOVA results.

Broad-sense heritability (H^2^b) ranged from moderate to very high across all traits (Table 2). Days to flowering showed the highest heritability (H^2^b = 0.99), followed closely by plant height (0.96), inflorescence length (0.90), 1000-seed weight (0.84), grain yield (0.83), and cross-seed area (0.79). These high heritability values (>0.75 for all traits) indicate that phenotypic selection would be effective for genetic improvement. Genetic advance as percentage of mean (GAM) revealed the expected gain from selection. Grain yield exhibited remarkably high GAM (117.54%), indicating that selecting the top-performing genotypes would result in more than doubling of mean yield. Inflorescence length (GAM = 52.63%) and plant height (GAM = 50.32%) also showed substantial expected gains, while days to flower (20.16%), and 1000-seed weight (38.77%) displayed moderate to high GAM values.

### 2.3. Trait Associations and Path Coefficient Analysis

**Figure 1:**
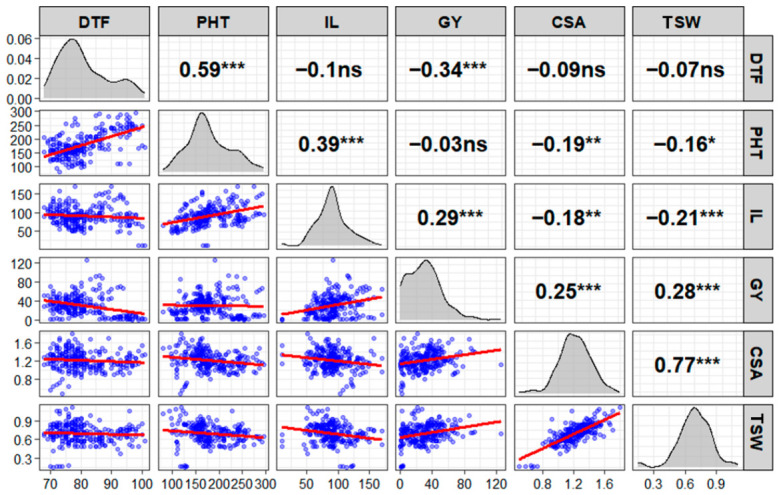
Phenotypic correlation matrix of six agronomic traits measured in 84 Amaranth (*Amaranthus* spp.) genotypes evaluated under irrigated conditions. The upper diagonal panels display Pearson correlation coefficients with significance levels: *** *p* < 0.001, ** *p* < 0.01, * *p* < 0.05, ns = not significant. The lower diagonal panels show scatterplots with linear regression lines (red) indicating the direction and strength of relationships. Diagonal panels present density distributions for each trait. Trait abbreviations: DTF = days to flower, PHT = plant height (cm), IL = inflorescence length (cm), GY = grain yield (g plant^−1^), CSA = cross-seed area (mm^2^), TSW = 1000-seed weight (g).

Phenotypic and genotypic correlation analyses revealed complex interrelationships among the six agronomic traits (Figure 1 and Figure S2), thereby providing insights into trait combinations that could facilitate or constrain simultaneous selection. Days to flowering showed significant negative phenotypic correlation with grain yield (*r* = −0.34, *p* < 0.001; Figure 1). This relationship was even stronger at the genotypic level (*r* = −0.36, *p* < 0.001; Figure S2). Days to flowering also exhibited significant positive correlations with plant height (*r* = 0.59, *p* < 0.001).

Plant height demonstrated significant positive correlations with inflorescence length at both phenotypic (*r* = 0.39, *p* < 0.001) and genotypic (*r* = 0.39, *p* < 0.01; Figure S2) levels, indicating that taller plants develop longer inflorescences. However, plant height showed no significant direct correlation with grain yield. Inflorescence length exhibited the strongest positive correlation with grain yield among all traits (phenotypic *r* = 0.29, *p* < 0.001; genotypic *r* = 0.28, *p* < 0.01), establishing inflorescence development as a key determinant of yield potential. This trait also correlated positively with plant height. Cross-seed area and 1000-seed weight were strongly and positively correlated with each other (phenotypic *r* = 0.77, *p* < 0.001; genotypic *r* = 0.82, *p* < 0.001), as expected for related seed size parameters. Both traits showed significant positive correlations with grain yield, confirming that larger seeds contribute positively to overall yield.

**Figure 2:**
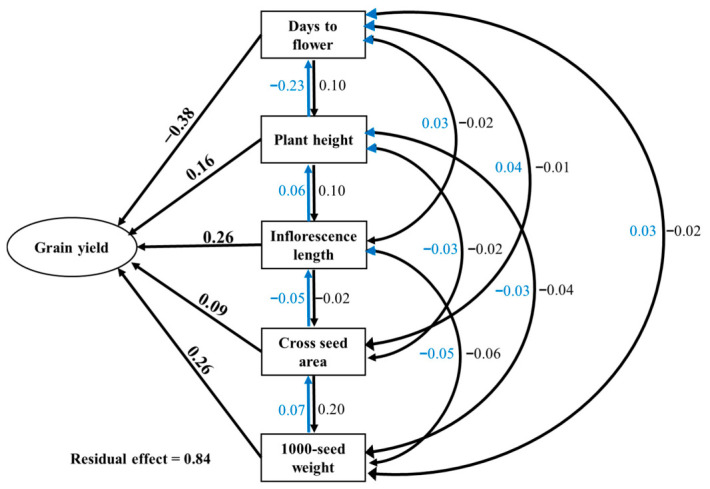
Phenotypic path analysis showing direct and indirect effects (path coefficients) of five agronomic traits on grain yield based on phenotypic data from 84 Amaranth genotypes under irrigated conditions. Numbers adjacent to arrows are standardized path coefficients. The bold number represents the direct effect of each trait on the grain yield. The other numbers represent the indirect effect of each trait on the grain yield via other independent traits. Blue and black color numbers and arrows show the direction of indirect effect of the trait via other traits on the grain yield. The residual effect is 0.84.

Path coefficient analysis partitioned the correlation coefficients into direct and indirect effects to identify traits with genuine influence on grain yield versus those whose correlations arise indirectly through other traits (Figure 2; Table S2). Multicollinearity diagnostics confirmed acceptable levels for all traits (VIF < 3, tolerance > 0.40; Table S3), ensuring stability of path coefficients. Phenotypic path analysis (Figure 2) revealed that 1000-seed weight exerted the strongest positive direct effect on grain yield (0.26), despite its moderate phenotypic correlation (*r* = 0.28). Inflorescence length showed a substantial positive direct effect (0.26) on grain yield, consistent with its significant positive correlation (*r* = 0.29). Days to flower exhibited a strong negative direct effect (−0.38) on grain yield, even stronger than its phenotypic correlation (*r* = −0.34), confirming that early flowering directly enhances yield rather than acting through correlated traits. Plant height displayed direct effect (0.16) on grain yield despite its correlation with inflorescence length, indicating that its apparent association with yield operates entirely through inflorescence development.

Genotypic path analysis (Table S2) confirmed these patterns, with 1000-seed weight (0.29), inflorescence length (0.25), and days to flower (−0.43) showing the strongest direct effects at the genetic level. The residual effect (0.84) indicates that approximately 16% of the variation in grain yield was explained by the five traits included in the model, suggesting that additional unmeasured factors contribute to yield determination.

### 2.4. Stepwise Multiple Regression for Yield Prediction

Stepwise multiple regression analysis validated the path analysis findings and established a predictive model for grain yield based on the most influential traits (Table 3). The stepwise procedure selected four traits as significant predictors, entering them in the following order: days to flowering entered first, explaining 11% of the yield variation (partial R^2^ = 0.11, *p* < 0.0001) with a negative parameter estimate (−0.94), confirming that early flowering enhances yield. Inflorescence length entered second, adding 6% to the explained variation (partial R^2^ = 0.06, *p* < 0.0001) with a positive parameter estimate (0.21), consistent with its role as a key yield component. One thousand-seed weight entered third, contributing 10% to the model (partial R^2^ = 0.10, *p* < 0.0001) with the largest positive parameter estimate (44.14), underscoring its strong direct influence on yield. Plant height entered fourth, adding only 1% to the explained variation (partial R^2^ = 0.01, *p* = 0.0854) with marginal significance, confirming its limited direct contribution to yield.

The final predictive model was:

Y = 45.24 − 0.94 (DTF) + 0.21 (IL) + 44.14 (TSW) + 0.06 (PHT)

where DTF = days to flowering, IL = inflorescence length, TSW = 1000-seed weight, and PHT = plant height.

The cumulative model R^2^ of 0.28 indicates that these four traits collectively explain 28% of the variation in grain yield, consistent with the path analysis residual effect. Days to flower, inflorescence length, and 1000-seed weight emerged as the three most important predictors, together accounting for 27% of the yield variation, while plant height contributed negligibly.

### 2.5. Multivariate Outlier Detection via Mahalanobis D^2^ Analysis

**Figure 3:**
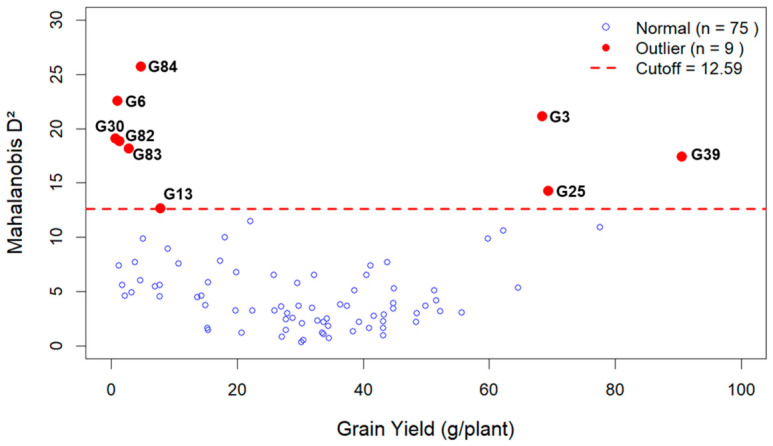
Scatter plot showing the relationship between grain yield and Mahalanobis D^2^ distance for 84 Amaranth genotypes. Blue circles = normal genotypes (*n* = 75); red circles = outlier genotypes (*n* = 9); red dashed line = critical value (χ^2^ = 12.59, df = 6, α = 0.05). Outlier genotypes are labeled. G39 (GY = 90.5 g/plant) is the top breeding candidate. The critical threshold and outlier classification were consistent between original and transformed grain yield data.

Mahalanobis distance (D^2^) analysis based on six quantitative traits identified nine genotypes as multivariate outliers (D^2^ > χ^2^ = 12.59, df = 6, α = 0.05), representing genetically distinct accessions with unique trait combinations (Table 4; Figure 3 and Figure S3).

The outlier genotypes, ranked by D^2^ value, were G84 (D^2^ = 25.72, *p* < 0.001) characterized by exceptionally long inflorescences (152.0 cm) and large seeds; G6 (D^2^ = 22.57, *p* < 0.001) notable for late flowering (100 days) and extremely short inflorescence (10.0 cm); G3 (D^2^ = 21.12, *p* < 0.01) combining tall stature (229.0 cm), long inflorescences (139.0 cm), and high yield (68.30 g/plant); G30 (D^2^ = 19.09, *p* < 0.01) with late flowering and very low yield (0.60 g/plant); G82 (D^2^ = 18.89, *p* < 0.01) exhibiting early flowering and small seeds; G83 (D^2^ = 18.18, *p* < 0.01) also showing small seeds and low yield; G39 (D^2^ = 17.47, *p* < 0.01) as the top breeding candidate with the highest yield among outliers (90.50 g/plant), tall stature (237.5 cm), long inflorescences (116.0 cm), and large seeds; G25 (D^2^ = 14.29, *p* < 0.05) as high-yielding (69.30 g/plant) with large seeds; and G13 (D^2^ = 12.65, *p* < 0.05) as extremely tall (282.5 cm) with moderate yield (Table 4).

The scatter plot of grain yield versus Mahalanobis distance (Figure 3) revealed that outlier status was associated with both exceptionally high-yielding (G39, G25, G3) and extremely low-yielding genotypes. Notably, G39 emerged as the most promising breeding candidate, combining outstanding yield with desirable traits including tall stature, long inflorescence, and large seeds, while maintaining perfect stability across replicates (GY_CV = 0). These results corroborate the path analysis findings and provide a practical selection index: selection for early flowering, long inflorescences, and heavy seeds to maximize grain yield in Amaranth breeding programs.

### 2.6. Principal Component Analysis and Promising Genotype Selection

Principal component analysis (PCA) reduced the six agronomic traits to three principal components with eigenvalues >1.0, collectively explaining 85.2% of the total morphological variation (Figure S4). PC1 accounted for 37.7% of the variance and was primarily associated with plant height and inflorescence length, representing plant architecture and reproductive potential. PC2 explained 24.5% of the variance and was strongly loaded with cross-seed area and 1000-seed weight, representing seed size characteristics. PC3 contributed 23.0% of the variance and was associated with days to flower and grain yield, representing phenology and productivity.

**Figure 4:**
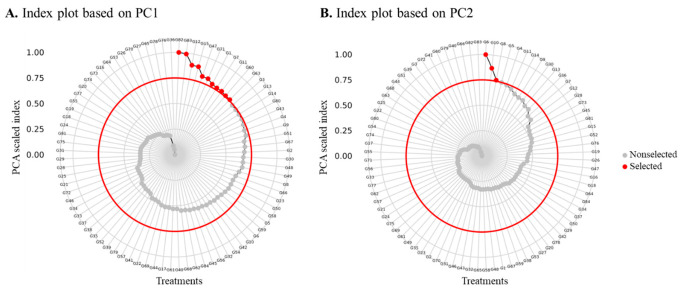
Index plots of the (**A**) first principal component (PC1) and (**B**) second principal component (PC2) for 84 Amaranth genotypes evaluated under irrigated conditions, based on six agronomic traits. Index scores were standardized to a 0–1 scale for direct comparability. The red line marks the 75% cutoff threshold. Genotypes above the 75th percentile (top 25%) are shown as red circles (promising genotypes), and genotypes below as gray circles (non-selected). The selected genotypes for PC1 and PC2 are presented in Supplementary Table S4.

Based on index scores standardized to a 0–1 scale, genotypes exceeding the 75th percentile (top 25%) were identified as promising for each principal component (Figure 4; Table S4). PC1-selected genotypes (*n* = 11) exhibited superior plant architecture, characterized by exceptional height and inflorescence development, including G12 (scaled index = 0.89) at 286.5 cm tall with long inflorescence, G15 (scaled index = 0.89) at 236.0 cm, and G82 (scaled index = 1.00) showing extreme inflorescence length (108.0 cm) relative to stature. PC2-selected genotypes (*n* = 3) exhibited superior seed size characteristics, with G6 (scaled index = 1.00) having large seeds (1.34 mm^2^), G8 (scaled index = 0.76) with large seeds (1.28 mm^2^), and G10 (scaled index = 0.87) possessing the largest seeds among the selected genotypes (1.38 mm^2^, 0.83 g/1000).

Integration with the outlier analysis revealed that three outlier genotypes were also identified among the PC-selected promising genotypes: G82 and G83 as top performers in PC1, and G6 as the top performer in PC2. Notably, G39, the top-yielding outlier, showed balanced performance across components but did not exceed the 75th percentile threshold in either PC1 or PC2 individually.

### 2.7. Molecular Diversity: Haplotype Network Analysis

**Figure 5:**
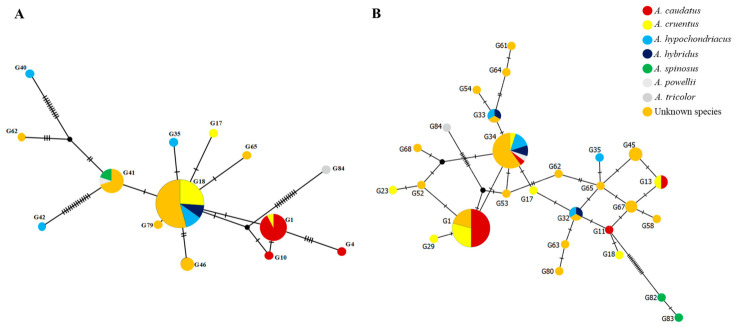
Median-joining networks based on DNA barcode sequences of 84 Amaranth genotypes. (**A**) *psbA-trnH* chloroplast DNA spacer region. (**B**) Nuclear internal transcribed spacer (ITS, including ITS1, 5.8S, and ITS2). Different colors represent different *Amaranthus* species: dark red = *A. caudatus*, yellow = *A. cruentus*, light blue = *A. hypochondriacus*, dark blue = *A. hybridus*, green = *A. spinosus*, gray = *A. powellii*, light gray = *A. tricolor*, and orange = unknown species. Circle size corresponds to the number of individuals sharing the same haplotype. Dashes along the connections represent single mutational steps (whereas black circles indicate haplotypes not observed in this study). The networks were constructed using PopART and edited using Microsoft PowerPoint.

The median-joining networks based on two DNA barcode markers, the intergenic spacer region separating the conserved *psbA* and *trnH* plastidic genes (chloroplast DNA) and the nuclear internal transcribed spacer (ITS, including ITS1, 5.8S, and ITS2), revealed the genetic relationships and haplotype distribution among the 84 Amaranth genotypes (Figure 5A,B).

The *psbA-trnH* chloroplast DNA network resolved multiple distinct haplotypes, with circle size proportional to haplotype frequency (Figure 5A). Several haplotypes were shared among multiple accessions, while others were unique to individual genotypes. Mutational steps between haplotypes revealed the evolutionary relationships and genetic distances among accessions. *A. caudatus* accessions formed a distinct cluster with limited haplotype diversity, suggesting a relatively narrow genetic basis within the cultivated grain Amaranth group. *A. cruentus* and *A. hypochondriacus* accessions showed greater haplotype dispersion, indicating broader genetic diversity. Unknown species were distributed across multiple haplotypes, some clustering with known species (suggesting putative taxonomic affinities) and others occupying distinct positions (representing potentially novel genetic groups).

The nuclear ITS DNA barcode network provided additional insights into the genetic diversity and relationships among the 84 Amaranth genotypes (Figure 5B). The ITS network revealed distinct haplotype groupings, with circle sizes reflecting the frequency of each haplotype within the germplasm collection. Multiple accessions shared common haplotypes, while others displayed unique haplotypes, indicating varying levels of genetic relatedness. *A. caudatus* accessions clustered together with limited haplotype variation, confirming the narrow genetic base observed in the chloroplast analysis. *A. cruentus* and *A. hypochondriacus* accessions were distributed across multiple haplotypes, reflecting broader genetic diversity within these species. Unknown species accessions were dispersed throughout the network, with some grouping alongside known species (providing clues for taxonomic assignment) and others occupying distinct positions (representing potentially unique genetic lineages). *A. hybridus*, *A. powellii*, *A. spinosus*, and *A. tricolor* occupied peripheral positions in the network, consistent with their genetic distinctiveness.

## 3. Discussion

### 3.1. Amaranth Genotypes Display Extensive Phenotypic Variation

Analysis of variance revealed highly significant differences (*p* < 0.01) among the 84 Amaranth genotypes for all six agronomic traits, confirming substantial genetic variability within the germplasm panel. These findings align with previous studies documenting significant genotypic variation in Amaranth collections from Ethiopia [14], Malawi [24], and exotic vegetable Amaranth genotypes [31]. The highly significant genotypic effects, coupled with non-significant block effects for most traits, indicate that observed variation reflects true genetic differences rather than environmental artifacts, thereby providing a reliable foundation for genetic improvement.

Productivity potential showed the widest variation, ranging from very low to exceptionally high values with a high coefficient of variation, comparable to findings from Singh [32], Yeshitila et al. [14] and Sefasi et al. [24]. The presence of genotypes at both extremes of the distribution provides breeders with valuable materials for understanding yield determination and direct selection. Architectural traits also displayed remarkable variation, with plant stature ranging from dwarf to tall types and inflorescence characteristics varying from extremely short to exceptionally long, consistent with reports from Yeshitila et al. [14] and Sefasi et al. [24]. Yeshitila et al. [15] similarly reported extensive variation in plant height among Ethiopian Amaranth genotypes.

Phenological traits showed relatively lower variation, suggesting tighter genetic control within cultivated Amaranth. Nevertheless, the observed range provides sufficient variation for adaptation to different photoperiod regimes and growing seasons [15,33]. The negative association between flowering time and productivity indicates that early-maturing genotypes tend to be more productive, likely due to their ability to complete grain filling before terminal stresses.

The wide ranges and CV values confirm that this germplasm collection captures significant genetic diversity, which is essential for sustained genetic gain through selection. The inclusion of multiple species and unknown accessions further enriches the genetic base, offering opportunities for interspecific hybridization and introgression of desirable traits [16,17].

### 3.2. High Heritability Enables Effective Selection for Key Traits

Genetic parameter estimates revealed high broad-sense heritability for all six agronomic traits, indicating that a substantial proportion of the phenotypic variation is attributable to genetic factors and would therefore respond favorably to selection. Similar high heritability estimates have been reported in Amaranth by Shrivastav et al. [20], Yadav et al. [13], Venkatesh et al. [34], and Patial et al. [35]. The high heritability coupled with low environmental influence suggests that phenotypic selection would be effective for genetic improvement. The highest heritability was observed for phenological traits, indicating strong genetic control with minimal environmental modification. This finding is consistent with observations reported by other researchers [13,35,36,37]. The combination of high heritability and a strong negative association with grain yield suggests that selection for early-flowering genotypes would be an effective yield improvement strategy.

Architectural traits also showed high heritability values, in agreement with Yeshitila et al. [14]. The combination of high heritability and high genetic advance for these traits indicates additive gene action and a favorable response to selection. Similarly, high genetic advance for morphological traits in Amaranth variability studies has been reported by Oduwaye et al. [37], Yadav et al. [13], and Patial et al. [35]. Grain yield exhibited high heritability coupled with remarkably high genetic advance, a combination particularly favorable for breeding programs as it indicates additive gene control and substantial potential for genetic gain. The combination of high heritability and high genetic advance for productivity and architectural traits suggests these traits are governed by additive gene action and would respond favorably to selection, thereby emerging as the most promising candidates for direct genetic improvement in Amaranth breeding programs.

### 3.3. Grain Yield Is Determined by Flowering Time, Inflorescence Length and Seed Weight

Understanding the interrelationships among traits and their effects on productivity is essential for developing efficient selection strategies in Amaranth breeding programs. Correlation and path coefficient analyses revealed that productivity is primarily determined by three key traits: phenology, inflorescence development, and thousand-seed weight.

A significant negative association with productivity was observed for phenological traits, indicating that earlier-maturing genotypes tend to produce higher yields [38,39]. Path analysis confirmed that this relationship is due to strong direct effects rather than indirect influences. Under the temperate conditions of Germany, where this study was conducted, temperatures drop considerably after August, and late-flowering genotypes experience cold stress during flowering and grain filling, which negatively impacts fertilization, seed set, and ultimately yield. Early-maturing genotypes complete their reproductive cycle before the onset of cold conditions, directly enhancing their yield potential. Similar findings have been reported by Henderson et al. [40], Artemyeva et al. [41], Dmitrieva and Ivanov [42], and Kanbar et al. [7].

From a physiological perspective, early flowering in Amaranth confers a significant adaptive advantage under temperate conditions. Flowering and grain filling are highly sensitive to low temperatures, which can disrupt pollen viability, reduce fertilization success, and limit seed set and development [43,44]. Early-maturing genotypes complete their reproductive cycle before the onset of cold stress, thereby avoiding these physiological constraints. Ecologically, this strategy represents a classic stress-escape mechanism, allowing plants to synchronize their life cycle with favorable seasonal windows [45]. Amaranth originated in warm tropical and subtropical regions of the Americas, and early-flowering genotypes are therefore better pre-adapted to temperate growing seasons such as those in Central Europe. This ecological adaptation has direct implications for breeding: early flowering should be prioritized when developing Amaranth varieties for temperate or high-latitude environments, whereas longer-season genotypes may be more suitable for tropical conditions where temperature is not limiting.

The strongest positive association with productivity was exhibited by inflorescence characteristics, with path analysis revealing substantial direct effects. This indicates that inflorescence development is a primary determinant of yield, functioning as the major sink structure where seeds develop. Longer inflorescences provide greater surface area for seed production. This findings are consistent with observations reported by other researchers [13,20,24,37,38].

The strongest positive direct effect on productivity was exerted by thousand-seed weight, despite its moderate phenotypic correlation. Path analysis revealed that the true contribution of thousand-seed weight is partially masked by opposing indirect effects through other traits, underscoring the importance of path analysis in revealing genuine trait relationships. Oduwaye et al. [37] and Venkatesh et al. [46] similarly found that thousand-seed weight had a high positive direct effect on grain yield, while Akther et al. [47] reported its significance in genetic divergence studies.

No significant direct association with productivity was observed for plant height, with path analysis revealing that its apparent relationship with yield operates entirely through inflorescence development, as documented by Yeshitila et al. [39], Shukla and Singh [48], and Venkatesh et al. [46].

Stepwise multiple regression corroborated these findings, with phenology, inflorescence length, and thousand-seed weight emerging as the three most important predictors. The identification of early maturity, long inflorescences, and heavy seeds provides breeders with potential selection criteria. However, the residual effect of 0.84 indicates that these three traits collectively explain only 16% of the variation in grain yield (path analysis) or 27% (stepwise regression). The majority of yield variation (72–84%) therefore remains unexplained by the model, suggesting that other unmeasured factors, such as seed number per inflorescence, branching architecture, harvest index, or physiological traits, likely contribute substantially to grain yield. Therefore, while early flowering, long inflorescences, and heavy seeds appear to be positive contributors, they should not be considered the definitive or sole determinants of yield. The absence of antagonistic relationships among these traits suggests that simultaneous improvement is feasible, thereby allowing the development of ideotypes combining all three characteristics. The importance of early maturity, long inflorescences, and thousand-seed weight as key yield determinants has been demonstrated across multiple *Amaranthus* species using stepwise multiple regression and path analysis [35,37,46,49]. However, these studies also emphasized that selection criteria should be species-specific: thousand-seed weight and stem diameter were effective for *A. hypochondriacus*, while thousand-seed weight and number of leaves were more suitable for *A. cruentus*.

### 3.4. Promising Genotypes Outperform the Germplasm Collection

Multivariate approaches including Mahalanobis D^2^ analysis and PCA enabled the detection of nine outlier genotypes with distinct phenotypic profiles and selection of promising accessions for specific trait complexes, consistent with approaches used by Sefasi et al. [24] and Yeshitila et al. [50]. Among the outliers, G39 emerged as the most promising breeding candidate, combining outstanding grain yield with tall stature, long inflorescence, and large seeds. This yield considerably exceeds those previously reported genotypes [32,35]. Two additional high-yielding outliers, G3 and G25, exhibited favorable trait combinations representing alternative ideotypes for different production systems. The exceptional performance of these three genotypes makes them ideal candidates for direct use as parents in hybridization programs or for potential release as varieties following further multi-location testing.

Principal component analysis provided a complementary framework for the selection of promising genotypes. PC1, representing plant architecture, identified 11 genotypes as valuable donors for improving inflorescence development. PC2, representing seed size characteristics, identified three genotypes possessing the largest seeds in the collection.

Similar PCA applications for trait identification and elite genotype selection in grain *Amaranthus* have been reported [24,39]. The limited overlap between D^2^ outliers and PC-selected elites reflects their different objectives: D^2^ identifies genotypes with extreme combinations across all traits, while PCA targets those excelling in specific trait complexes. This explains why G39, despite top yield, did not exceed the 75th percentile in individual PCs; its superiority derives from optimal combination of traits rather than from the extreme expression of any single trait.

The field experiment was conducted at a single location (Karlsruhe, Germany) during one growing season (2020). Consequently, the heritability estimates reported reflect variance components under these specific conditions and may not be transferable to other environments without accounting for genotype × environment (G × E) interaction. Similarly, the identification of G39 as a high-yielding genotype does not guarantee its performance under different soil types, climate conditions, or management practices. Therefore, the term “promising” is used here to denote superior performance under the conditions tested, not as a validated recommendation for release. Trials across multiple seasons and locations are essential to assess G × E interactions and confirm the stability of G39 and other candidate genotypes (G3, G25) before any breeding or variety release decisions can be made.

### 3.5. Phenotypic and Molecular Data Together Guide Amaranth Breeding

The integration of phenotypic and molecular data in the present study provides a comprehensive framework for developing efficient breeding strategies in Amaranth. The complementary nature of these approaches offers breeders multiple entry points for genetic improvement: morphological characterization for trait evaluation, genetic parameter estimation for understanding inheritance patterns, multivariate analyses for genotype selection, and DNA barcoding for assessing genetic relationships.

The close phylogenetic relationships and the tendency for hybridization pose limitations to the resolution of barcoding markers. However, the individual SNPs required to interchange between two given genotypes are informative and can improve resolution, because statistical measures of difference are complemented by specific patterns, comparable to a fingerprint. This strategy has been widely used to infer evolutionary relationships between closely related taxa under the designation criterion of specific quality [51]. We made use of this strategy by inferring haplotype networks based on chloroplast *psbA-trnH* and nuclear ITS sequences that revealed distinct genetic relationships among the 84 Amaranth genotypes and provided molecular evidence for the complex domestication history of the genus. Clear separation of the major grain Amaranth species was observed, with *A. caudatus* accessions forming a compact cluster with limited haplotype diversity, suggesting a relatively narrow genetic base within this cultivated species. This finding aligns with Jamalluddin et al. [19], who reported reduced genetic diversity in cultivated Amaranth species due to domestication bottlenecks.

Greater haplotype dispersion was observed for *A. cruentus* and *A. hypochondriacus* accessions, indicating broader genetic diversity within these species. This pattern reflects the complex domestication history of Amaranth, a crop considered incompletely domesticated. Amaranth was domesticated independently twice in the Americas: once in Peru, giving rise to *A. caudatus* (*kiwicha*) from the wild ancestor *A. quitensis*, and once in Mexico, producing *A. cruentus* and *A. hypochondriacus* from the common progenitor *A. hybridus* [1,6]. Following these independent domestication events, there was subsequent gene flow and exchange between these centers, further complicating the genetic architecture of cultivated forms. Thus, when researchers or breeders refer to “Amaranth,” they may be working with genetically distinct crop types originating from different ancestral species with unique evolutionary trajectories.

Clear differences were observed between the two marker systems. The nuclear ITS network exhibited clearer separation and greater haplotype diversity than the chloroplast *psbA-trnH* network, due to fundamental differences in inheritance patterns. Nuclear markers are biparentally inherited, capturing genetic contributions from both parents and providing a comprehensive view of evolutionary relationships. In contrast, chloroplast DNA is maternally inherited, tracing only seed-mediated gene flow and maternal lineages. The higher mutation rate and faster evolution of nuclear ribosomal DNA make ITS particularly informative for distinguishing closely related accessions and revealing recent evolutionary divergence.

Building on our previous study using the same germplasm [12], which focused on species authentication, the present study revealed a notable concordance between phenotypic and molecular diversity. The nine genotypes identified as multivariate outliers by Mahalanobis D^2^ analysis, including the top-yielding candidate G39, consistently occupied unique or peripheral positions in both chloroplast and nuclear ITS haplotype networks. This convergence provides independent validation that phenotypic distinctiveness corresponds to genuine genetic differentiation, a pattern similarly observed in integrated studies of other crops such as rice [52,53], sorghum [54], sugarcane [55], wheat [56], and Brassica [57]. Compared to these studies, our work uniquely combines multiple multivariate techniques (path analysis, stepwise regression, D^2^, PCA) with two complementary DNA barcode systems (chloroplast and nuclear) within a single germplasm collection, offering a more comprehensive framework for linking phenotypic performance to genetic identity. This integrated approach is particularly valuable in *Amaranthus*, where morphological plasticity and frequent hybridization complicate species identification based solely on visual traits.

It is acknowledged that the *psbA-trnH* and ITS markers used in this study have known limitations in resolving closely related species in certain plant groups. However, these markers remain among the most informative and widely used barcodes for plants [58,59,60,61,62,63,64]. In our previous work using the same markers [12], we successfully identified a species-specific Single Nucleotide Polymorphism (SNP) in the *psbA-trnH* region that separated *A. caudatus* from all other *Amaranthus* species, enabling the development of a sequencing-free Amplified Refractory Mutation System (ARMS) authentication assay. This demonstrates that, despite general limitations, these markers were informative for our germplasm collection. Nevertheless, higher-resolution genotyping (e.g., SSR markers, SNP arrays) would be required for definitive taxonomic assignment of the 38 unknown accessions. Therefore, our haplotype assignments for unknown accessions are presented as provisional and should be interpreted with caution.

The 38 accessions of unknown taxonomic status were distributed across multiple haplotypes, with some clustering alongside known species and others occupying distinct positions. This pattern provides initial clues for taxonomic assignment and highlights the value of molecular markers for resolving taxonomic uncertainties in *Amaranthus*, a genus where morphological identification is often challenging due to phenotypic plasticity and ongoing gene flow between domesticated and weedy forms [65]. The integration of phenotypic and molecular diversity in this study is primarily qualitative; a quantitative approach, such as a Mantel test correlating phenotypic and genetic distance matrices, would provide a more rigorous assessment and is recommended for future studies.

## 4. Materials and Methods

### 4.1. Plant Materials

A total of 84 Amaranth genotypes were evaluated (Table 5), consisting of 15 *A. caudatus*, 14 *A. cruentus*, 9 *A. hypochondriacus*, 4 *A. hybridus*, 2 *A. spinosus*, 1 *A. tricolor*, 1 *A. powellii*, and 38 genotypes of uncertain taxonomic classification. The germplasm encompasses commercially utilized species, landraces, and agriculturally bred varieties, thereby capturing a substantial portion of the genetic diversity currently in human use. All accessions were raised to flowering and their taxonomic identities were determined following digital documentation of floral traits and plant habitus according to taxonomic keys of the Flora of China (http://www.efloras.org/florataxon.aspx?flora_id=2&taxon_id=101257) (accessed on 13 March 2000), the Flora of North America (http://www.efloras.org/florataxon.aspx?flora_id=1&taxon_id=101257) (accessed on 13 March 2000) [66].

### 4.2. Greenhouse Planting and Field Transplanting

A field experiment was conducted under temperate conditions at the Botanical Garden of Karlsruhe Institute of Technology (KIT), Germany (49°0′24.8004″ N, 8°24′13.1508″ E; 119 m above sea level), characterized by an oceanic climate with mean temperatures ranging from −1 °C in winter to 26 °C in summer. On 15 April 2020, surface-sterilized seeds of all 84 accessions were sown in 100-well trays filled with a 1:1:1 mixture of peat moss, perlite, and soil. Six seeds were planted per well and later thinned to one seedling per well after germination, with 50 seedlings raised per accession in the greenhouse at 25 ± 3 °C under a 12 h photoperiod. Three weeks post-germination, seedlings were transplanted to the field in a randomized complete block design with three replications. Each plot measured 2.5 m in length with row spacing of 0.65 m and plant spacing of 0.40 m within rows. Each block contained three rows per accession; the two outer rows served as borders, while data were collected from plants in the middle row. Based on soil analysis, organic fertilizer (Hauert Hornoska^®^ Special) at 100 g/m^2^ and NPK at 90:60:40 kg/ha were applied. Soil moisture was maintained at 70–80% field capacity through irrigation until seed maturity.

### 4.3. Phenotyping and Morphological Evaluation

For morphological observations, three plants were randomly selected from the center of the second row in each plot. This sample size is consistent with standard protocols for Amaranth germplasm characterization, where three to five representative plants per replication are commonly used to balance statistical reliability with practical feasibility [33,67]. Data recorded included days to flower, plant height (cm), inflorescence length (cm), grain yield (g/plant), 1000-seed weight (g), and seed cross area (mm^2^). Inflorescence length was measured from the lowest to the highest flower on each plant. Seed cross area was determined from three randomly chosen seeds per plant sample in each replicate. Individual seeds were imaged at a fixed orientation using a Keyence VHX-950F digital microscope (Keyence, Neu-Isenburg, Germany), and area measurements were obtained from digital images using ImageJ 1.53 Version software (https://imagej.net/ij/; accessed on 11 January 2023). At full maturity, panicles were hand-harvested with pruning shears, dried in a hoop house for two weeks, and manually threshed. The extracted seeds were winnowed and evaluated for grain yield and 1000-seed weight.

### 4.4. DNA Extraction, PCR Amplification and Sequencing

Genomic DNA of the 84 Amaranth accessions was extracted following the CTAB protocol described in our previous work [12]. Briefly, 100 mg of frozen leaf tissue was ground, incubated with CTAB extraction buffer containing β-mercaptoethanol at 65 °C for one hour, followed by chloroform-isoamyl alcohol purification and isopropanol precipitation. The extracted DNA was dissolved in nuclease-free water with RNase A, and its concentration and purity were assessed spectrophotometrically.

Two DNA barcode markers were amplified by PCR: the chloroplast intergenic spacer region separating the *psbA* and *trnH* genes, and the nuclear internal transcribed spacer (ITS) comprising ITS1, 5.8S, and ITS2 (See Table S5). Each 30 µL PCR reaction contained 20.4 µL nuclease-free water, 3 µL Thermopol Buffer, 3 µL bovine serum albumin, 0.6 µL dNTPs, 0.6 µL each of forward and reverse primers (200 nM), 0.3 µL Taq polymerase, and 1.5 µL template DNA (50 ng/µL). Thermal cycling conditions for both markers are detailed in Table S5. Amplification products were visualized by gel electrophoresis using 1.5% agarose stained with Midori Green, with fragment sizes determined against a 100 bp DNA ladder. PCR products were purified using the MSB Spin PCRapace Kit prior to sequencing, which was outsourced to Macrogen Europe B.V. (Amsterdam, The Netherlands) and Eurofins (Ebersberg, Germany). Sequence quality was assessed using FinchTV Version 1.4.01 software, and all sequences were deposited in the NCBI database.

### 4.5. Data Processing

Mean value, standard error, maximum value, minimum value, range, skewness, kurtosis, and coefficient of variation of 6 measurable morphological traits were calculated using R studio software (V4.5.2). These descriptive statistics provide a basic understanding of data distribution and variability, which is fundamental for subsequent in-depth analyses. Before the analysis, the data were subjected to normality testing to ensure conformity to basic assumptions of analysis of variance (ANOVA) [68]. Grain yield and 1000-seed weight data were transformed using the square root transformation method in R studio software (V4.5.2). Consequently, two-way ANOVA (genotype and block) was performed under a randomized complete block design (RCBD) to evaluate the significance of differences among genotypes [69]. The significance of differences between means was determined at *p* < 0.05 using the Tukey’s test [70].

The phenotypic variance (σ^2^p), genotypic variance (σ^2^g), phenotypic coefficient of variation (PCV), and genotypic coefficient of variation (GCV) were computed as per Burton [71]. Broad-sense heritability (h^2^b) was estimated using the formula described by [72]. Genetic advance (GA) was calculated by the formula suggested by Lush [73]. Genetic advance as a percent of the mean (GA%) was assessed as defined by Johnson et al. [74].

Phenotypic and genotypic correlations were estimated using the standard procedure suggested by Miller et al. [75] from the corresponding variance and covariance components. Significant correlation coefficients were tested at (*n* − 2) degrees of freedom on a ‘t’ table from Fisher and Yates at 5% and 1% significance levels. Simple phenotypic and genotypic correlation coefficients were separated into direct and indirect effects using path analysis [21].

Before proceeding with path analysis, the data were examined for multicollinearity using the variance inflation factor (VIF) and tolerance statistics. This step is critical in path analysis to ensure the stability and accuracy of the estimated path coefficients. Small tolerance values (much lower than 0.1) or high VIF values (>10) show high multicollinearity [76]. Following the confirmation of non-severe multicollinearity, phenotypic and genotypic path analyses were carried out using RAISINS (R and AI Solutions for INferential Statistics [77]) to study the direct and indirect contributions of traits to the associations. Stepwise multiple regression analysis was carried out as a confirmatory tool to validate the path analysis findings and to assess the combined contribution of the selected traits to grain yield [78].

To identify multivariate outliers among the 84 Amaranth accessions, Mahalanobis distance squared ($D^{2}$) analysis was performed based on six agronomic traits evaluated under irrigated conditions [22]. Mahalanobis distance measures the multivariate distance of each genotype from the centroid of the trait space, accounting for correlations among traits. Under multivariate normality, the $D^{2}$ values follow a chi-square ($\chi^{2}$) distribution with degrees of freedom equal to the number of traits (df = 6). Genotypes with $D^{2}$ values exceeding the critical chi-square value at α=0.05 ($\chi^{2}=12.59$) were classified as multivariate outliers. The analysis was conducted using R studio software (V4.5.2).

To examine the agronomic significance of multivariate outlier status, a scatter plot was constructed illustrating the relationship between grain yield and Mahalanobis $D^{2}$ distance. For each of the 84 Amaranth genotypes evaluated under irrigated conditions, the Mahalanobis $D^{2}$ value (derived from six agronomic traits) was plotted on the abscissa (*x*-axis) against grain yield (g/plant) on the ordinate (*y*-axis). The scatter plot was generated using R software (version 4.2.5) with the ggplot2 package.

Principal component analysis (PCA) was conducted to reduce the dimensionality of the dataset and elucidate patterns of morphological variation among 84 Amaranth genotypes. The analysis was based on six quantitative traits. PCA was performed using the correlation matrix to standardize the variables and account for differences in measurement scales. The eigenvalues were calculated for each principal component (PC), and the proportion of total variance explained by each PC was determined. Components with eigenvalues greater than 1.0 were retained for interpretation based on the Kaiser criterion [79], and a scree plot was constructed to visualize the decreasing contribution of successive components to the total variance [80]. Following principal component analysis, index plots were constructed for the first two principal components (PC1 and PC2) to visualize the distribution of 84 Amaranth genotypes based on their multivariate phenotypic profiles. For each genotype, the PC1 and PC2 scores were extracted from the PCA conducted on six quantitative traits evaluated under irrigated conditions. To enable direct comparability between components and to facilitate genotype selection, the PC scores were standardized to a 0–1 scale using the following normalization formula:

$$\mathrm{Normalized}\;\mathrm{score}=\frac{\mathrm{Actual}\;\mathrm{score}-\mathrm{Minimum}\;\mathrm{score}}{\mathrm{Maximum}\;\mathrm{score}-\mathrm{Minimum}\;\mathrm{score}}$$

This transformation rescales the original PC scores to the unit interval [0, 1], where values close to 1 indicate genotypes with high positive contributions to the respective principal component, and values near 0 represent genotypes with low or negative contributions [81]. All PCA computations and visualizations were performed using RAISINS (R and AI Solutions for Inferential Statistics).

To examine genetic relationships among the 84 Amaranth genotypes, haplotype networks were constructed using *psbA-trnH* chloroplast and ITS sequences. Sequences were aligned and exported in Nexus format. The median-joining network algorithm was employed to infer evolutionary relationships [29]. The network was generated using PopART version 1.7 with default parameters, including epsilon = 0 (no star contraction) [82]. The networks display unique haplotypes as circles proportional to their frequency, mutational steps as connecting lines with hash marks, and inferred intermediate haplotypes (median vectors) as small black nodes.

## 5. Conclusions

This study demonstrates that substantial genetic diversity exists among the 84 Amaranth genotypes, with grain yield showing the widest variation and high heritability estimates indicating a favorable response to selection. Path analysis identified early flowering, long inflorescences, and heavy seeds as the primary determinants of grain yield, providing breeders with a focused selection index for indirect improvement. Multivariate approaches enabled the identification of nine outlier genotypes with distinct phenotypic profiles, among which G39 emerged as the most promising breeding candidate combining exceptional yield with desirable architecture and large seeds, while principal component analysis further identified complementary promising donors for plant architecture and seed size characteristics. Molecular characterization using chloroplast and nuclear markers validated these findings, with outlier genotypes occupying unique haplotype positions and the nuclear ITS network providing clearer resolution due to biparental inheritance, confirming that phenotypically distinct genotypes like G82, G83, G13, G10, and G39 possess unique genetic combinations. The integration of phenotypic and molecular data provides breeders with well-characterized germplasm, validated selection criteria, and prioritized parental materials for Amaranth improvement programs. Further multi-location and multi-season evaluations are recommended to ensure the stability and adaptability of these promising germplasm accessions.

## Figures and Tables

**Table 1:** Descriptive statistics for six agronomic traits measured in 84 Amaranth (*Amaranthus* spp.) genotypes evaluated under irrigated conditions with three replications (*n* = 252 observations per trait).

Statistic	Mean ± SE	Min.	Max.	Range	Skewness	Kurtosis	CV%
Days to flowering (day)	80.82 ± 0.41	68.00	101.00	33.00	0.72	2.54	0.87
Plant height (cm)	179.13 ± 5.08	80.00	295.00	215.00	0.44	2.66	4.91
Inflorescence length (cm)	90.96 ± 4.59	10.00	170.00	160.00	0.26	4.00	8.74
Grain yield (g/plant)	30.14 ± 4.97	0.25	125.56	125.32	0.83	4.37	28.55
Cross-seed area (mm^2^)	1.22 ± 0.05	0.50	1.79	1.29	−0.20	3.67	7.70
1000-seed weight (g)	0.69 ± 0.04	0.15	1.11	0.96	−0.61	5.02	9.00

SE = Standard Error of the mean; CV = Coefficient of Variation (%).

**Table 2:** Genetic parameters for six agronomic traits in 84 Amaranth (*Amaranthus* spp.) genotypes evaluated under irrigated conditions (*n* = 252 observations per trait).

Statistic	σ^2^g	σ^2^p	GCV (%)	PCV (%)	H^2^b (%)	GA	GAM
Days to flowering	63.08	63.58	9.83	9.87	0.99	16.30	20.16
Plant height	1989.25	2066.72	24.90	25.38	0.96	90.14	50.32
Inflorescence length	597.08	660.26	26.86	28.25	0.90	47.87	52.63
Grain yield	357.12	431.18	62.70	68.89	0.83	35.43	117.54
Cross-seed area	0.03	0.04	15.00	16.86	0.79	0.33	27.49
1000-seed weight	0.02	0.02	20.56	22.46	0.84	0.27	38.77

σ^2^p = phenotypic variance; σ^2^g = genotypic variance; GCV = genotypic coefficient of variation (%); PCV = phenotypic coefficient of variation (%); H^2^b = broad-sense heritability; GA = genetic advance; GAM = genetic advance as a percentage of the mean (%).

**Table 3:** Relative contribution (partial and model R^2^), *F* value, and probability values for predicting Amaranth grain yield by the stepwise procedure analysis (*n* = 84).

Step	Variable Entered	Partial R^2^	Model R^2^	Parameter Estimate	Standard Error	*p* > *F*
1	Days to flowering	0.11	0.11	−0.94	0.18	<0.0001
2	Inflorescence length	0.06	0.17	0.21	0.05	<0.0001
3	1000-seed weight	0.10	0.27	44.14	7.38	<0.0001
4	Plant height	0.01	0.28	0.06	0.03	0.0854

**Table 4:** Multivariate outlier genotypes identified by Mahalanobis D^2^ analysis in 84 Amaranth genotypes based on six agronomic traits evaluated under irrigated conditions.

Rank	Genotype ID	Mahalanobis D^2^	*p* Value	Significance	DTF	GY	PHT	IL	CSA	TSW	GY_CV
1	G84	25.72	0.0003	***	88.00	4.70	163.00	152.00	1.42	0.76	0
2	G6	22.57	0.0010	***	100.00	0.90	170.50	10.00	1.34	0.57	0
3	G3	21.12	0.0017	**	93.00	68.30	229.00	139.00	0.94	0.71	0
4	G30	19.09	0.0040	**	93.70	0.60	110.50	48.00	1.13	0.44	0
5	G82	18.89	0.0044	**	71.70	1.20	121.00	108.00	0.58	0.16	0
6	G83	18.18	0.0058	**	90.00	2.80	128.50	121.50	0.69	0.17	0
7	G39	17.47	0.0077	**	86.00	90.50	237.50	116.00	1.43	0.71	0
8	G25	14.29	0.0266	*	78.30	69.30	156.50	95.00	1.10	0.87	0
9	G13	12.65	0.0489	*	77.00	7.80	282.50	93.50	1.37	0.63	0

DTF = days to flower (days), GY = grain yield (g/plant), PHT = plant height (cm), IL = inflorescence length (cm), CSA = cross-seed area (mm), TSW = 1000-seed weight (g), GY_CV = coefficient of variation for grain yield (%). Significance: *** *p* < 0.001, ** *p* < 0.01, * *p* < 0.05. Critical value = 12.59. Genotypes were ranked according to D^2^ values. GY_CV = 0 indicates perfect stability across replicates. The critical threshold and outlier classification were consistent between original and transformed grain yield data.

**Table 5:** List of Amaranth genotypes used in the study, including species designation, number of accessions per species, and genotype identifiers.

Species Name	No. of Genotypes	Name of Genotypes
*A. caudatus*	15	G1 (*A. caudatus*_7469), G2 (*A. caudatus*_3807), G3 (*A. caudatus*_8102), G4 (*A. caudatus*_8600), G5 (*A. caudatus* cv. Beige_3707), G6 (*A. caudatus* cv. Fox_Peter 4_8299), G7 (*A. caudatus* cv. Marron_2496), G8 (*A. caudatus* cv. Negro_9442), G9 (*A. caudatus* cv. Oscar Blanco 1 _9444), G10 (*A. caudatus* cv. Oscar Blanco 2 _9445), G11 (*A. caudatus* cv. Oscar Blanco 3_9425), G12 (*A. caudatus* cv. Peter 1_8300), G13 (*A. caudatus* cv. Peter 2_8301), G14 (*A. caudatus* cv. Peter 3_8302), G15 (*A. caudatus* cv. Rosado_9408)
*A. cruentus*	14	G16 (*A. cruentus* cv. Andy_8055), G17 (*A. cruentus*_7470), G18 (*A. cruentus* cv. A 10_8043), G19 (*A. cruentus* cv. Amar_8091), G20 (*A. cruentus* cv. Anna_8081), G21 (*A. cruentus* cv. Bärnkrafft_8080), G22 (*A. cruentus* cv. K 266_8037), G23 (*A. cruentus* cv. K 436_8040), G24 (*A. cruentus* cv. K72_8049), G25 (*A. cruentus* cv. MT3_8041), G26 (*A. cruentus* cv. Nu World_8042), G27 (*A. cruentus* cv. Puerto Moutt_8056), G28 (*A. cruentus* cv. Suvarna_8046), G29 (*A. cruentus* cv. Villarica_8057)
*A. hybridus*	4	G30 (*A. hybridus*_8939), G31 (*A. hybridus* cv. Pastewny_8052), G32 (*A. hybridus* cv. Turkiestan_8053), G33 (*A. hybridus* cv. Ural_8054)
*A. hypochondriacus*	9	G34 (*A. hypochondriacus*_8104), G35 (*A. hypochondriacus* cv. Anderer Typ_8085), G36 (*A. hypochondriacus* cv. Anderer Typ_8096), G37 (*A. hypochondriacus* cv. K343_8038), G38 (*A. hypochondriacus* cv. K343 bunt_8083), G39 (*A. hypochondriacus* cv. Mittlerer Typ (8084), G40 = *A. hypochondriacus* cv. Mittlerer Typ OR dunkel_8095), G41 (*A. hypochondriacus* cv. Mittlerer Typ OR hell_8090), G42 (*A. hypochondriacus* x *hybridus* cv. K432_8039)
*A. powellii*	1	G43 (*A. powellii*_8938)
Unknown species	38	G44 (A. species_8079), G45 (A. species_8601), G46 (A. species cv. Arma_8082), G47 (A. species cv. Arora 2_8298), G48 (A. species cv. C2_8060), G49 (A. species cv. C3_8061), G50 (A. species cv. C4_8044), G51 (A. species cv. C5_8062), G52 (A. species cv. C6_8045), G53 (A. species cv. Energietyp_8094), G54 ( A. species cv. K10_8063), G55 (A. species cv. K25_8064), G56 (A. species cv. K39_8065), G57 (A. species cv. K41_8066), G58 (A. species cv. K42_8067), G59 (A. species cv. K47_8068), G60 (A. species cv. K48_8069), G61 (A. species cv. K50_8070), G62 (A. species cv. K51_8071), G63 (A. species cv. K53_8072), G64 (A. species cv. K61_8047), G65 (A. species cv. K62_8073), G66 (A. species cv. K63_8048), G67 (A. species cv. K64_8074), G68 (A. species cv. K67_8058), G69 (A. species cv. K71_8075), G70 (A. species cv. K78_8050), G71 (A. species cv. K80_8076), G72 (A. species cv. K88_8077), G73 (A. species cv. K91_8051), G74 (A. species cv. Mittlerer Typ Rot_8089), G75 (A. species cv. Mittlerer Typ Rot_8093), G76 (A. species cv. Neuer Typ_8087), G77 (A. species cv. Neuer Typ_8092), G78 (A. species cv. NTCX_8088), G79 (A. species cv. Poleski Ukrai_8078), G80 (A. species cv. Sierra Leone_8059), G81 (A. species cv. Typ X_8086)
*A. spinosus*	2	G82 (*A. spinosus*_3809), G83 (*A. spinosus*_8290)
*A. tricolor*	1	G84 (*A. tricolor*_8103)

## Data Availability

Sequences were uploaded to the NCBI database. All data supporting the findings of this study are available within the paper and within its Supplementary Materials published online. Any other data are available from the corresponding author A.K. upon request.

## Supplementary Materials

The following supporting information can be downloaded at: https://www.mdpi.com/article/10.3390/plants15101493/s1, Table S1. Genotypic means (± standard error) and ANOVA summary for six agronomic traits in 84 Amaranth (*Amaranthus* spp.) genotypes evaluated under irrigated conditions in a randomized complete block design with three replications (*n* = 252 observations per trait); Table S2. Genotypic path coefficient analysis showing direct (bold diagonal) and indirect effects of five agronomic traits on grain yield in 84 Amaranth (*Amaranthus* spp.) genotypes under irrigated conditions; Table S3. Variance Inflation Factor (VIF) and Tolerance values for five agronomic traits in Amaranth (*Amaranthus* spp.) under irrigated conditions; Table S4. Selected genotypes based on PC1 and PC2 index scores from principal component analysis of 84 Amaranth genotypes evaluated under irrigated conditions; Table S5. Primers used to amplify DNA markers and the amplification protocol; Figure S1. Frequency distributions of six key agronomic traits in 84 Amaranth (*Amaranthus* spp.) genotypes evaluated under irrigated conditions; Figure S2. Genotypic correlation matrix of six quantitative traits measured in 84 Amaranth (*Amaranthus* spp.) genotypes evaluated under irrigated conditions; Figure S3. Evaluation of multivariate distances; Figure S4. Scree plot showing the percentage of variance explained by each principal component (PC1 to PC6) from principal component analysis of six agronomic traits in 84 Amaranth genotypes evaluated under irrigated conditions. Refs. [83,84,85] are cited in Supplementary Materials.
