# Supplementary material for: Integrating Multivariate Analysis and DNA Barcoding for Amaranth Germplasm Characterization and Promising Genotype Selection
Source: Plants (Basel). 2026 May 13;15(10):1493. doi: 10.3390/plants15101493 (PMC13210587; doi:10.3390/plants15101493)
Supplement: Supplementary file 1 [file plants-15-01493-s001.zip › plants-4270990-supplementary.pdf]

Supplementary materials

Integrating Multivariate Analysis and DNA Barcoding for Amaranth Germplasm Characterization and Promising Genotype Selection

Adnan Kanbar, Yaman Jabbour, Peter Nick

List of Supplemental Figures/Tables:

Figure S1. Frequency distribution of six key agronomic traits in 84 amaranth (*Amaranthus* spp.) genotypes evaluated under irrigated conditions.

Figure S2. Genotypic correlation matrix of six traits measured in 84 Amaranth (*Amaranthus* spp.) genotypes evaluated under irrigated conditions.

Figure S3. Evaluation of multivariate distances.

Figure S4. Scree plot showing the percentage of variance explained by each principal component (PC1 to PC6) from principal component analysis of six agronomic traits in 84 Amaranth genotypes evaluated under irrigated conditions.

Table S1. Genotypic means (± standard error) and ANOVA summary for six agronomic traits in 84 Amaranth (*Amaranthus* spp.) genotypes evaluated under irrigated conditions in a randomized complete block design with three replications (n = 252 observations per trait).

Table S2. Genotypic path coefficient analysis showing direct (bold diagonal) and indirect effects of five agronomic traits on grain yield in 84 amaranth (*Amaranthus* spp.) genotypes under irrigated conditions.

Table S3. Variance Inflation Factor (VIF) and Tolerance values for five agronomic traits in amaranth (*Amaranthus* spp.) under irrigated conditions.

Table S4. Selected genotypes based on PC1 and PC2 index scores from principal component analysis of 84 Amaranth genotypes evaluated under irrigated conditions.

Table S5. Primers used to amplify DNA markers and the amplification protocol.

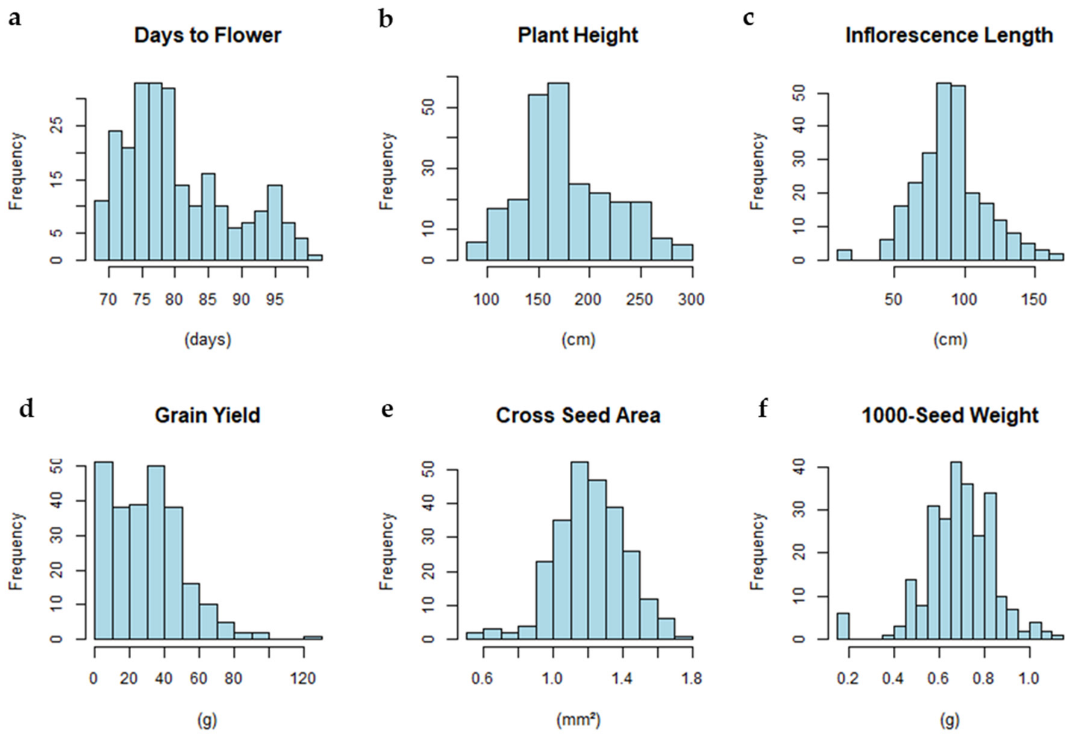

Figure S1. Frequency distribution of six agronomic traits in 84 Amaranth (*Amaranthus* spp.) genotypes evaluated under irrigated conditions. Histograms illustrate phenotypic variation for (a) days to flower, (b) plant height (cm), (c) inflorescence length (cm), (d) grain yield (g), (e) cross seed area (mm^2), and (f) 1000-seed weight (g). Data represent means of three replications in a randomized complete block design (n = 252). The continuous distribution and wide variation observed for all traits indicate substantial genetic diversity, providing scope for selection in breeding programs.

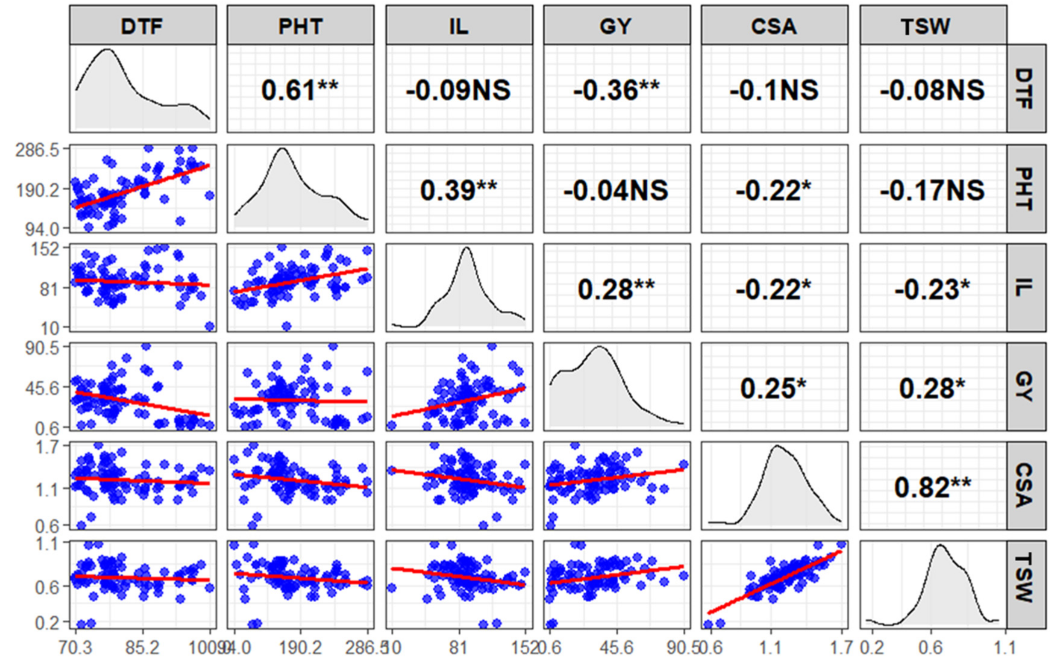

Figure S2. Genotypic correlation matrix of six traits measured in 84 Amaranth (*Amaranthus* spp.) genotypes evaluated under irrigated conditions. The upper diagonal panels display Pearson correlation coefficients with significance levels: ** $p<0.01$, * $p<0.05$, NS = not significant. The lower diagonal panels show scatterplots with linear regression lines (red) indicating the direction and strength of relationships. Diagonal panels present density distributions for each trait. Trait abbreviations: DTF = days to flower, PHT = plant height (cm), IL = inflorescence length (cm), GY = grain yield (g plant^{-1}), CSA = cross-sectional seed area (mm^2), TSW = 1000-seed weight (g).

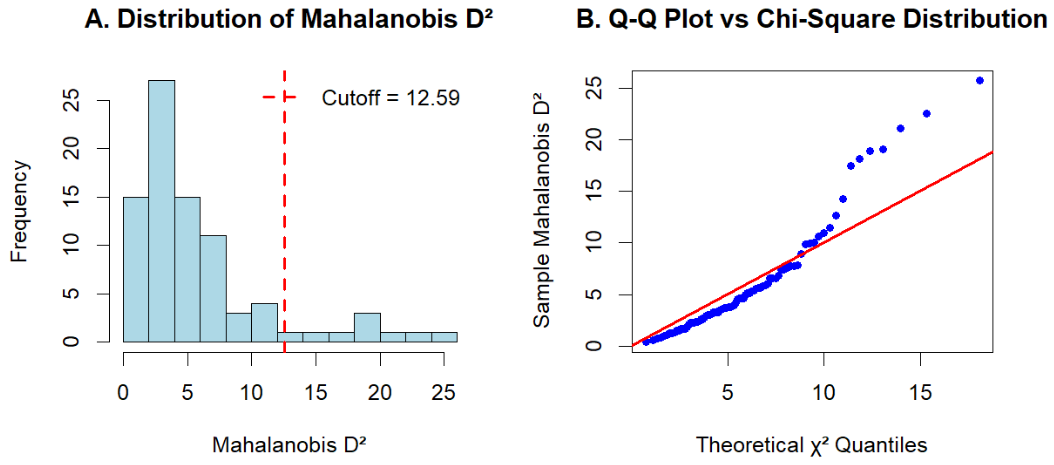

Figure S3. Evaluation of multivariate distances. (A) Histogram of Mahalanobis D^2 values. (B) Q-Q plot comparing observed D^2 values against the expected chi-square distribution.

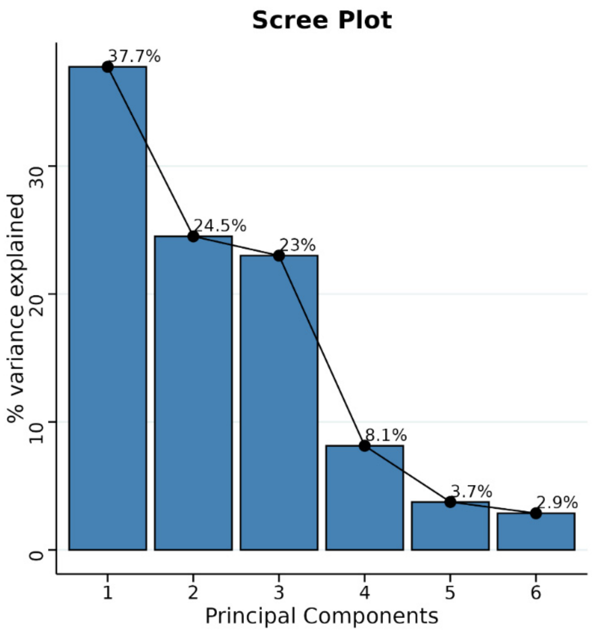

Figure S4. Scree plot showing the percentage of variance explained by each principal component (PC1 to PC6) from principal component analysis of six agronomic traits in 84 Amaranth genotypes evaluated under irrigated conditions. The first three principal components collectively explained 85.2% of the total morphological variation (PC1: 37.7%, PC2: 24.5%, PC3: 23.0%). The remaining components (PC4–PC6) accounted for 14.8% of the variation (PC4: 8.1%, PC5: 3.7%, PC6: 2.9%).

Table S1. Genotypic means (± standard error) and ANOVA summary for six agronomic traits in 84 Amaranth (*Amaranthus* spp.) genotypes evaluated under irrigated conditions in a randomized complete block design with three replications (n = 252 observations per trait).

Genotyppe	DTF	PHT	IL	GY	CSA	TSW
G1	85.33±0.58	205.00± 5.00	88.50± 3.50	27.85± 3.96	1.00±0.05	0.48±0.00
G2	88.67±1.53	210.00±30.00	112.50± 4.50	44.81± 0.40	1.19±0.04	0.57±0.00
G3	93.00±1.00	229.00± 9.00	139.00± 4.00	68.35± 2.79	0.94±0.03	0.71±0.02
G4	95.67±1.53	240.50±13.50	76.50± 8.50	1.16± 0.92	1.13±0.04	0.76±0.29
G5	94.67±1.53	210.00± 5.00	64.00± 0.00	7.71± 2.84	1.23±0.11	0.76±0.02
G6	100.00±1.00	170.50± 4.50	10.00± 0.00	0.90± 0.54	1.34±0.20	0.57±0.10
G7	89.00±2.65	241.50± 1.50	81.00± 2.00	2.10± 0.93	1.11±0.07	0.62±0.04
G8	96.67±1.53	242.50± 2.50	77.50± 7.50	7.64± 1.24	1.28±0.02	0.78±0.02
G9	96.67±1.53	242.50± 2.50	93.00± 2.00	6.87± 1.67	1.20±0.07	0.75±0.02
G10	98.33±2.08	242.00± 8.00	66.00± 4.00	3.69± 0.01	1.38±0.05	0.83±0.02
G11	96.00±1.00	254.50± 1.50	80.50± 5.50	1.71± 0.52	1.20±0.11	0.66±0.08
G12	96.33±1.53	286.50± 8.50	97.50± 2.50	10.70± 0.92	1.04±0.09	0.65±0.00
G13	93.33±1.53	279.50± 7.50	93.50± 3.50	7.77± 0.17	1.37±0.09	0.62±0.01
G14	93.67±2.08	255.00± 7.00	79.50± 3.50	4.54± 3.35	1.15±0.03	0.72±0.03
G15	88.67±2.08	236.00± 4.00	84.50± 7.50	3.17± 1.20	0.93±0.02	0.51±0.02
G16	77.33±1.53	166.50± 1.50	85.50± 9.50	33.67± 4.30	1.48±0.06	0.86±0.06
G17	76.67±2.08	174.50± 2.50	88.00± 6.00	43.10± 7.16	1.08±0.02	0.68±0.01
G18	72.67±2.08	149.00± 8.00	71.50± 1.50	22.32± 1.92	1.31±0.02	0.80±0.01
G19	80.67±2.08	150.50± 5.50	59.00± 2.00	41.63± 9.19	1.33±0.07	0.80±0.01
G20	78.33±1.53	163.00± 2.00	90.00± 2.00	43.10± 7.70	1.39±0.05	0.87±0.02
G21	78.67±2.08	150.00± 3.00	84.00± 6.00	55.64± 0.14	1.24±0.03	0.76±0.06
G22	71.33±1.53	173.00±14.00	101.50±15.50	34.21± 0.73	1.14±0.10	0.68±0.10
G23	78.67±2.08	167.50± 5.50	78.00± 1.00	31.81± 8.91	0.96±0.08	0.59±0.01
G24	72.67±2.08	160.00±12.00	95.00±13.00	34.38±12.82	1.34±0.14	0.80±0.01
G25	78.33±1.53	156.50± 3.50	95.00± 5.00	69.34± 0.98	1.10±0.18	0.86±0.06
G26	76.67±2.08	144.50±10.50	66.50± 0.50	37.42± 2.07	1.52±0.04	0.82±0.02
G27	78.33±1.53	160.00± 1.00	87.50± 3.50	52.20±12.39	1.52±0.15	0.88±0.04
G28	78.67±2.08	103.50± 3.50	49.50± 3.50	13.62± 5.21	1.27±0.14	0.72±0.03
G29	79.67±2.08	165.00± 2.00	83.00± 4.00	40.93± 4.83	1.31±0.14	0.84±0.02
G30	93.67±2.08	110.50± 0.50	48.00± 3.00	0.61± 0.04	1.13±0.09	0.44±0.08
G31	71.67±2.08	126.50± 1.50	89.50± 4.50	19.76± 7.39	1.21±0.01	0.84±0.01
G32	77.00±2.65	155.00± 7.00	89.50± 3.50	15.37± 3.25	1.10±0.02	0.62±0.03
G33	70.33±2.08	151.50± 4.50	89.50± 0.50	28.71± 4.20	1.28±0.03	0.69±0.01
G34	76.33±2.08	155.50±15.50	73.00± 3.00	27.67± 1.67	1.31±0.00	0.71±0.04
G35	85.67±1.53	196.00± 2.00	99.00± 1.00	64.54±10.22	1.27±0.13	0.79±0.12
G36	75.33±2.08	123.00± 5.00	55.00± 4.00	17.97± 3.10	1.70±0.10	1.06±0.05
G37	79.00±2.65	130.50±12.50	92.00± 2.00	19.68± 0.12	1.35±0.02	0.72±0.00
G38	77.33±2.08	175.50± 4.50	83.00± 6.00	34.51±10.04	1.28±0.14	0.72±0.02
G39	86.00±1.00	237.50±17.50	116.00± 2.00	90.50± 2.35	1.43±0.04	0.71±0.01
G40	70.33±2.08	205.00± 5.00	114.00±16.00	51.19± 9.28	1.12±0.11	0.66±0.01
G41	74.67±1.53	146.00± 5.00	134.00± 4.00	32.15± 0.45	1.27±0.00	0.67±0.03
G42	79.00±2.65	109.50± 0.50	60.00± 3.00	17.27± 1.95	0.93±0.08	0.56±0.00
G43	85.67±1.53	177.50±12.50	118.50± 8.50	14.29± 0.60	1.10±0.09	0.56±0.02
G44	81.67±1.53	200.00±10.00	86.00± 6.00	77.51±48.05	1.08±0.05	0.64±0.04
G45	84.33±2.08	205.00± 5.00	82.50± 8.50	15.19± 0.11	1.22±0.13	0.74±0.03
G46	77.33±2.08	161.50± 2.50	64.50± 1.50	49.81± 6.27	1.22±0.00	0.70±0.04
G47	93.33±2.08	227.50± 2.50	133.50± 6.50	4.97± 3.78	1.12±0.06	0.68±0.01
G48	84.33±2.08	210.00±10.00	96.00± 4.00	33.64±13.99	1.07±0.03	0.62±0.04
G49	79.67±1.53	175.50± 0.50	97.50± 7.50	20.70± 1.45	1.03±0.12	0.57±0.01
G50	85.67±1.53	173.00± 7.00	84.50± 5.50	30.28± 2.44	1.07±0.08	0.64±0.01
G51	77.33±2.08	256.50± 4.50	126.00± 4.00	40.49± 7.93	1.14±0.14	0.62±0.06
G52	79.00±1.00	119.00± 9.00	55.00± 1.00	14.94± 1.36	1.15±0.02	0.68±0.01
G53	76.67±2.08	159.50± 0.50	87.50± 7.50	44.69± 3.33	1.55±0.07	0.84±0.13

Genotyppe	DTF	PHT	IL	GY	CSA	TSW
G54	76.67±2.08	168.50± 8.50	106.50±12.50	27.71± 0.98	1.19±0.08	0.56±0.03
G55	71.67±2.08	157.50± 1.50	94.00± 6.00	41.11± 3.39	1.47±0.11	0.68±0.04
G56	80.67±2.08	207.50± 2.50	106.00± 2.00	43.14± 5.09	1.17±0.11	0.67±0.09
G57	76.67±2.08	158.50± 7.50	98.00± 1.00	33.48± 6.21	1.25±0.02	0.64±0.03
G58	81.33±1.53	182.50± 3.50	91.50± 0.50	27.00± 3.93	1.15±0.07	0.58±0.10
G59	83.67±2.08	192.50±17.50	100.50± 4.50	30.08±12.98	1.17±0.06	0.66±0.06
G60	86.67±2.08	286.00± 6.00	145.00± 5.00	62.23± 3.60	1.19±0.03	0.60±0.04
G61	76.67±2.08	168.50±11.50	89.00± 1.00	30.42±12.81	1.15±0.16	0.63±0.08
G62	78.67±2.08	175.50± 2.50	92.50±20.50	38.33± 7.18	1.17±0.09	0.59±0.10
G63	88.33±1.53	230.00± 9.00	148.00±22.00	43.82±20.28	1.09±0.19	0.62±0.12
G64	76.67±1.53	151.00± 1.00	74.00± 1.00	39.34± 2.82	1.46±0.02	0.82±0.05
G65	73.33±1.53	135.00± 8.00	91.50± 8.50	36.27± 2.19	1.53±0.13	0.86±0.02
G66	72.67±1.53	197.50±16.50	138.50±18.50	38.54± 8.42	1.07±0.28	0.63±0.10
G67	80.67±2.08	172.00± 5.00	62.50± 2.50	29.51±10.23	0.94±0.01	0.48±0.01
G68	70.67±1.53	185.00± 4.00	104.50± 5.50	29.73± 5.58	1.14±0.03	0.61±0.20
G69	73.00±1.00	159.00± 3.00	75.00± 3.00	26.96± 0.19	1.06±0.04	0.65±0.07
G70	72.67±2.08	126.50± 2.50	72.00± 5.00	48.44± 3.65	1.34±0.07	0.83±0.01
G71	78.33±1.53	247.50±27.50	130.00±40.00	8.92± 2.43	1.05±0.09	0.59±0.08
G72	70.67±2.08	192.00± 8.00	110.00±10.00	51.56±18.50	1.33±0.02	0.73±0.07
G73	77.67±2.08	98.50± 1.50	56.00± 6.00	15.36± 2.55	1.30±0.06	0.84±0.10
G74	73.67±2.08	164.00±12.00	101.50± 8.50	48.32± 5.89	1.42±0.02	0.81±0.02
G75	71.67±2.08	153.50± 3.50	88.00± 4.00	32.68± 0.63	1.36±0.01	0.74±0.02
G76	73.33±1.53	94.00±14.00	74.00± 9.00	22.07± 5.08	1.50±0.03	1.05±0.03
G77	76.33±1.53	181.50± 4.50	112.00± 5.00	44.70± 3.84	1.40±0.07	0.89±0.05
G78	80.67±2.08	114.00± 2.00	84.00± 2.00	59.80±26.20	1.60±0.07	0.91±0.07
G79	71.67±2.08	159.50±19.50	117.00±12.00	43.23±11.40	1.26±0.06	0.65±0.04
G80	74.33±1.53	211.50±11.50	93.00± 2.00	25.77± 6.62	0.98±0.01	0.47±0.02
G81	78.67±2.08	150.00± 6.00	53.00± 3.00	25.89± 3.02	1.28±0.11	0.81±0.03
G82	71.67±2.08	121.00± 5.00	108.00± 5.00	1.24± 0.38	0.58±0.08	0.16±0.01
G83	73.67±2.08	128.50± 1.50	121.50± 1.50	2.75± 0.69	0.69±0.04	0.17±0.01
G84	90.00±2.65	163.00± 0.00	152.00± 4.00	4.67± 0.53	1.42±0.28	0.76±0.01
Grand mean	82.26	179.34	88.55	29.57	1.23	0.70
F-Test (Genotype)	**	**	**	**	**	**
F-Test (Block)	**	ns	ns	ns	ns	ns
HSD	2.49	31.04	28.04	30.35	0.35	0.00
MSE	0.50	77.48	63.19	74.06	0.01	0.00
CV(%)	0.87	4.91	8.74	28.55	7.70	9.00

DTF = days to flower (days), PHT = plant height (cm), IL = inflorescence length (cm), GY = grain yield (g plant^{-1}), CSA = cross seed area (mm^2), TSW = 1000-seed weight (g). *F*-test (genotype and block) from ANOVA, HSD = Tukey's honestly significant difference (α = 0.05), MSE = mean square error, CV(%) = coefficient of variation percentage. Significance levels: ** = $p < 0.01$, ns = non-significant.

Table S2. Genotypic path coefficient analysis showing direct (bold diagonal) and indirect effects of five agronomic traits on grain yield in 84 Amaranth (*Amaranthus* spp.) genotypes under irrigated conditions. Values were derived from genotypic correlation matrices based on genotype means across three replications. Bold values on the diagonal represent the direct effects (path coefficients) of each trait on grain yield. Off-diagonal values represent indirect effects of row traits on grain yield via column traits. The residual effect equal to 0.84.

Trait	Days to flower	Plant height	Inflorescence length	Cross seed area	1000-seed weight	Correlation with grain yield
Days to flower	**-0.433**	0.113	-0.024	-0.006	-0.023	-0.36**
Plant height	-0.266	**0.184**	0.097	-0.013	-0.053	-0.04ns
Inflorescence length	0.042	0.071	**0.251**	-0.014	-0.068	0.28**
Cross seed area	0.046	-0.044	-0.063	**0.056**	0.249	0.25*
1000-seed weight	0.035	-0.034	-0.059	0.048	**0.290**	0.28*

Significance levels: ** $p < 0.01$, * $p < 0.05$, ns = non-significant.

Table S3. Variance Inflation Factor (VIF) and Tolerance values for five agronomic traits in Amaranth (*Amaranthus* spp.) under irrigated conditions. VIF quantifies the severity of multicollinearity among predictor traits in the path analysis model, while Tolerance (1/VIF) indicates the proportion of variance in each trait not explained by other traits. VIF values < 5 (or Tolerance > 0.20) indicate acceptable multicollinearity, while VIF > 10 (Tolerance < 0.10) suggests serious multicollinearity concerns. All traits in the present study showed VIF values below 3, indicating no serious multicollinearity issues in the path analysis.

Trait	VIF	Tolerance
Days to flower	1.896	0.527
Plant height	2.235	0.447
Inflorescence length	1.506	0.664
Cross seed area	2.471	0.405
1000-seed weight	2.474	0.404

Table S4. Selected genotypes based on PC1 and PC2 index scores from principal component analysis of 84 Amaranth genotypes evaluated under irrigated conditions. The default cutoff selection is set at 75%; genotypes above this threshold (top 25%) are considered elite. Index scores were standardized to a 0–1 scale for direct comparability. PC1 selected genotypes (n=11) and PC2 selected genotypes (n=3) represent the top-performing accessions for traits associated with each principal component.

PC	Genotype	Index Score	Scaled Index	Days to flower	Plant height	Inflorescence length	Grain yield	Cross seed area	1000-seed weight
PC1	G1	1.85	0.78	85.33	205.00	88.50	27.85	1.00	0.48
	G7	1.79	0.77	89.00	241.50	81.00	2.10	1.11	0.62
	G11	1.79	0.77	96.00	254.50	80.50	1.71	1.20	0.67
	G12	2.65	0.89	96.33	286.50	97.50	10.70	1.04	0.65
	G15	2.66	0.89	88.67	236.00	84.50	3.17	0.93	0.51
	G47	2.05	0.81	93.33	227.50	133.50	4.97	1.12	0.68
	G60	1.70	0.76	86.67	286.00	145.00	62.23	1.19	0.60
	G63	1.76	0.76	88.33	230.00	148.00	43.82	1.09	0.62
	G71	2.05	0.81	78.33	247.50	130.00	8.92	1.05	0.59
	G82	3.42	1.00	71.67	121.00	108.00	1.24	0.58	0.16
	G83	3.33	0.99	73.67	128.50	121.50	2.75	0.69	0.17
PC2	G6	3.83	1.00	100.00	170.50	10.00	0.90	1.34	0.57
	G8	2.39	0.76	96.67	242.50	77.50	7.64	1.28	0.79
	G10	3.06	0.87	98.33	242.00	66.00	3.69	1.38	0.83

Table S5. Primers used to amplify DNA markers and the amplification protocol.

DNA marker	Primer	Sequence (5′...3′)	References	Amplification Protocol
ITS	ITS-5_F	GGAAGTAAAAGTCGTAACAAGG	(White et al. 1990)	95 ℃ 2 min; 94 ℃ 30 s, 56 ℃ 30 s, 68 ℃ 1 min, 35 cycles; 68 ℃ 5 min
	ITS-4_R	TCCTCCGCTTATTGATATGC		
psbA-trnH	psbA_F	GTTATGCATGAACGTAATGCTC	(Sang et al. 1997)	95 ℃ 2 min; 94 ℃ 1 min, 56 ℃ 30 s, 68 ℃ 45 s, 35 cycles; 68 ℃ 5 min
	trnH_R	CGCGCATGGTGGATTCACAATCC	(Tate and Simpson 2003)	
	ARMS_F	GCTCCTTTATTAGTACTAGTCTGG		

References

Sang, T.; Crawford, D.J.; Stuessy, T.F. Chloroplast DNA phylogeny, reticulate evolution, and biogeography of Paeonia (*Paeoniaceae*). *American Journal of Botany* 1997, 84, 1120–1136.

Tate, J.A.; Simpson, B.B. Paraphyly of Tarasa (*Malvaceae*) and diverse origins of the polyploid species. *Systematic Botany* 2003, 28, 723–737.

White, T.J.; Bruns, T.; Lee, S.; Taylor, J. Amplification and direct sequencing of fungal ribosomal RNA genes for phylogenetics. In *PCR Protocols: A Guide to Methods and Applications*; Innis, M.A., Gelfand, D.H., Sninsky, J.J., White, T.J., Eds.; Academic Press: San Diego, CA, USA, 1990; pp. 315–322.
